# Persistent Renal Dysfunction After Acute Kidney Injury Among STEMI Patients Undergoing Primary Coronary Intervention: Prevalence and Predictors

**DOI:** 10.1002/clc.70002

**Published:** 2024-10-08

**Authors:** Shir Frydman, Ophir Freund, Haytham Abu katash, Daniel Rimbrot, Shmuel Banai, Yacov Shacham

**Affiliations:** ^1^ Department of Cardiology, Tel‐Aviv Sourasky Medical Center Affiliated to the Sackler Faculty of Medicine Tel‐Aviv University Tel‐Aviv Israel

**Keywords:** AKD, AKI, predictors, prevalence, STEMI

## Abstract

**Background:**

Acute kidney injury (AKI) is a common and serious complication of ST‐elevation myocardial infarction (STEMI). AKI and chronic kidney disease (CKD) are highly heterogeneous, leaving a wide gap between them. Therefore, the term acute kidney disease (AKD) was implemented, describing prolonged renal injury between 7 and 90 days. We aimed to evaluate the prevalence and predictors of AKD among STEMI patients.

**Methods:**

This retrospective observational study included 2940 consecutive patients admitted with STEMI between 2008 and 2022. Renal function was assessed upon admission and routinely thereafter. Renal outcomes were evaluated according to KDIGO criteria, with AKD defined as persistent renal injury of between 7 and 90 days.

**Results:**

Two hundred and fifty‐two subjects with STEMI and AKI were included; of them, 117 (46%) developed AKD. Among baseline CKD patients, higher rates of AKD were observed (60% vs. 46%). KDIGO index ≥ 2 was an independent predictor for AKD in in subjects without baseline CKD (AOR 2.63, 95% CI 1.07−6.53). In subjects with baseline CKD, older age and higher creatinine were independent predictors for AKD. Subjects with AKD had a higher 1‐year mortality rate (HR 3.39, 95% CI 1.71−6.72, *p* < 0.01). This trend was mainly driven by the CKD subpopulation where higher mortality rates for AKD on CKD were observed (HR 5.26, 95% CI 1.83−15.1, *p* < 0.01).

**Conclusion:**

AKD is common among STEMI patients with AKI. The presence of CKD and higher KDIGO stage should prompt strict monitoring for early diagnosis, treatment, and prevention of renal function deterioration.

## Graphical abstract

Acute kidney disease (AKD) is an entity that refers to prolonged renal injury between 7 and 90 days. We aimed to evaluate the prevalence and predictors of AKD among 3000 STEMI patients and found that AKD is quite common (9%) and that is associated with 1‐year mortality. KDIGO stage, age, and peak creatinine were independently associated with AKD progression.

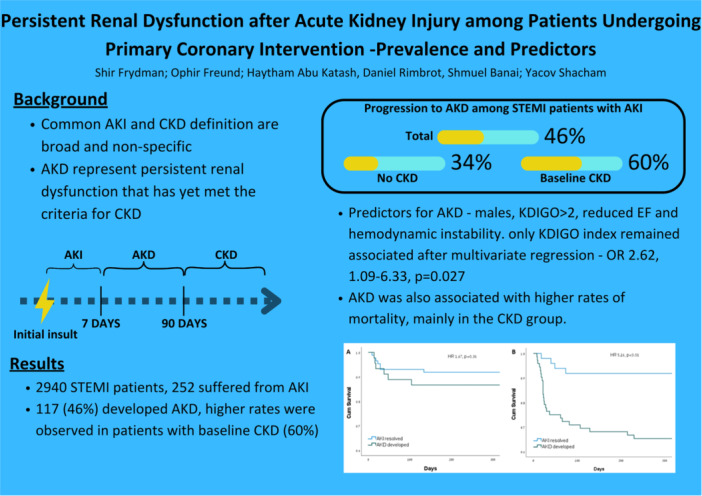

## 1 Introduction

Renal injury is a common and serious complication of acute coronary syndrome (ACS) in general, and of ST‐elevation myocardial infraction (STEMI) in particular [1].

Acute kidney injury (AKI) in coronary patients occurs through several proposed mechanisms, including contrast associated nephropathy, hemodynamic instability, renal perfusion compromise, and nephrotoxic medication, all of which are enhanced in STEMI subset population [2]. Subsequently, renal impairment was associated with adverse outcomes for STEMI patients in several large registries [3, 4]. Therefore, attempting to predict patients at risk for renal injury holds great promise for early treatment and prevention [5]. Common definitions for renal injury, including AKI and chronic kidney disease (CKD) are highly heterogeneous, leaving a wide gap between these two entities [6]. Hence, in an effort to better understand and classify renal injuries, the term acute kidney disease (AKD) was implemented—an entity that describes prolonged renal injury that persists beyond 7 days and up to 90 days following insult [7]. Non‐surprisingly, AKD was found to be associated with higher rates of CKD progression and 1‐year mortality [8, 9].

Most ACS patient with AKI will recover within short time, yet longer duration to said renal recovery was shown to be associated with adverse outcomes and mortality [10, 11]. Small scale reports attempted to address AKD in ACS patients, yet the specific more vulnerable population of STEMI patients was not studied before. We aimed to evaluate the prevalence of AKD in this unique population and identify predictors for its progression in a large cohort.

## 2 Methods

**Figure 1:**
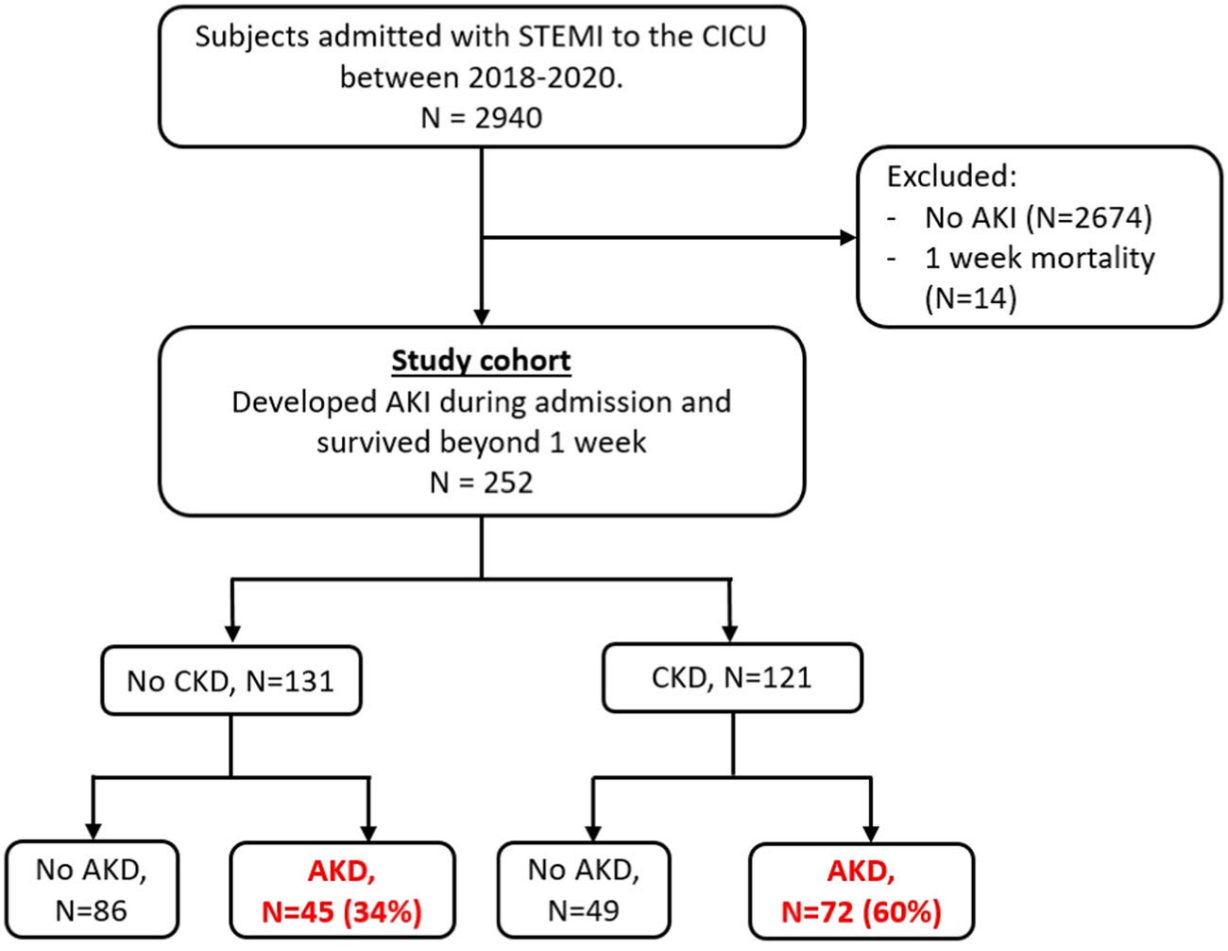
Study inclusion process.

This is a retrospective analysis based on a database including all patients that were admitted to the cardiac intensive care unit (CICU) of a single tertiary referral hospital with the diagnosis of acute STEMI between January 2008 and 2022. The hospital (Tel‐Aviv Sourasky Medical Center) has a 24/7 primary percutaneous coronary intervention services. From the initial database, patients were excluded if records of their renal function were missing (*n* = 25) or if they required chronic peritoneal or hemodialysis (*n* = 18). Following that and considering the aim of our study, patients with AKI (with either normal baseline kidney function or CKD) that survived beyond 7 days were considered as our study cohort and were assessed for the occurrence of AKD (Figure 1).

The diagnosis of STEMI was made if a typical history of chest pain occurred, with diagnostic electrocardiographic changes, and serial elevation of serum cardiac biomarkers [12]. The diagnosis was verified for each patient before inclusion to the database. PPCI was carried out in patients within 12 h from the onset of pain or 12−24 h from symptoms onset given ongoing significant pain. Treatment with statins, renin/angiotensin blockers, and β‐blockers were started in all patients unless contraindicated. Following PCI, 0.9% saline was given for 12 h at a rate of 1 mL/kg/h or lower if patients had an overt heart failure.

Baseline demographic and medical history, treatment characteristics, and laboratory results were included in the database for all patients (*n* = 2940). Left ventricular ejection fraction was measured within the first 48 h by a bedside echocardiography in all cases. In‐hospital mortality and complications occurring during the hospitalization were evaluated without missing data. Complications included acute renal failure, cardiogenic shock, or the need of intra‐aortic balloon pump (IABP), new‐onset ventricular tachycardia (VT)/fibrillation (VF) episodes, bleeding (requiring blood transfusion), and stent thrombosis.

The study was conducted according to the Declaration of Helsinki and approved by the Tel‐Aviv Sourasky medical center review board (TLV‐16‐0224). Informed consent was obtained from all subjects involved in the study.

### 2.1 Renal Outcomes

Serum creatine was regularly evaluated upon patient admission, before primary PCI, and at least once daily during hospitalization. The estimated glomerular filtration rate (eGFR) was calculated with the Chronic Kidney Disease Epidemiology Collaboration (CKD‐EPI) equation and a presenting eGFR of under 60 mL/min/1.73 m² was determined for CKD definition [13]. AKI and AKD were defined according to the “kidney disease: improving global outcomes (KDIGO)” criteria [14]. AKI was defined as an increase in serum creatinine by > 0.3 mg/dL in 2 days or by > 50% in 1 weak. Severe AKI was defined as peak creatinine higher than twice the admission creatinine level, which is correlated to Stages 2 and 3 of the KDIGO AKI staging [14]. AKD was defined as AKI or decrease in serum creatinine by ≥ 35% for more than a week, compared with baseline level and for less than 3 months.

### 2.2 Statistical Analysis

Data were first analyzed for the entire study cohort and compared between patients with and without AKD. Univariate and multivariate analyses for predictors of AKD were performed separately among patients with AKI alone and among patients with AKI on CKD. Multivariate analyses included variables from the univariate analysis that were associated with AKD (with *p* < 0.10) after evaluation of clinical relevance by the research team. The association between AKD and mortality was assessed by Kaplan−Meier analysis. Continuous data were presented as median (interquartile range [IQR]) and compared with the Mann−Whitney *U* test. Categorical data were presented as total (percentage) and compared with Chi‐square tests. Analyses were performed with SPSS software version 28.0 (SPSS Inc., Chicago, IL, USA), with *p* < 0.05 determined for significance.

## 3 Results

During the study period, 2940 subjects were admitted in the CICU with STEMI, 252 (9%) developed AKI during admission, survived beyond 7 days, and were included in our cohort (Figure 1) with a median follow‐up time of 23 (IQR 17−40) days. Of them, 135 (54%) had resolution of their AKI by Day 7, while 117 (46%) developed AKD. The finding was more prominent among patients with baseline CKD, of whom 52 (60%) developed AKD, compared to 45 (34%) in the normal baseline kidney function subpopulation. Comparison of baseline characteristics between the groups is presented in Table 1. Subjects with AKD were older, and had higher rates of hypertension, CKD, and requirement of ionotropic support during admission.

**Table 1:** Comparison of baseline and clinical characteristics between subjects with and without AKD.

Variable	AKI resolved *N* = 135 (%)	AKD developed *N* = 117 (%)	*p*
Age, median (IQR)	68 (60–77)	78 (64–85)	< 0.01
Female sex	36 (27)	28 (24)	0.62
Hyperlipidemia	80 (59)	63 (54)	0.39
Hypertension	84 (62)	93 (80)	< 0.01
Diabetes	49 (36)	43 (37)	0.94
Past myocardial infarction	31 (23)	26 (22)	0.90
Atrial fibrillation	18 (13)	22 (19)	0.24
Chronic kidney disease	49 (36)	72 (62)	< 0.01
Number of obstructive coronary artery vessels			
1	41 (30)	40 (34)	0.71
2	33 (24)	30 (26)	
3	61 (45)	47 (40)	
Door to balloon, min, median (IQR)	45 (30–70)	45 (30–60)	0.09
Ejection fraction, %, median (IQR)	45 (35–50)	40 (35–45)	0.13
Peri‐procedural complications			
Mechanical ventilation	28 (21)	24 (21)	0.96
Ventricular tachycardia/fibrillation	24 (18)	14 (12)	0.20
Bleeding	23 (17)	18 (15)	0.72
Inotropic use	16 (12)	27 (23)	0.02
Peak creatinine (mg/dL), median (IQR)	1.60 (1.40−2.06)	1.98 (1.45−2.80)	< 0.01
Peak C‐reactive protein, median (IQR)	84 (33−163)	102 (19−177)	0.90

Given the strong association between AKD development and CKD, and the differences in etiologies, predictors for AKD were analyzed separately based on the presence of CKD (Table 2). Univariate analysis in subjects without CKD (Table 2) found that AKI with KDIGO severity above 1, ejection fraction below 40%, and ionotropic use were associated with AKD development. In multivariate analysis, KDIGO severity above 1 remained an independent predictor for AKD (adjusted OR 2.63, 95% CI 1.07−6.53, *p* = 0.037), while female gender showed a trend as an independent predictor for lower rates of AKD (AOR 0.36, 95% CI 0.12−1.04, *p* = 0.056).

**Table 2:** Univariate and multivariate analyses for predictors of AKD in subjects with and without CKD.

	Variable	Univariate		Multivariate	
		OR (95% CI)	*p*	Adjusted OR	*p*
No CKD	Female sex	0.34 (0.12−0.97)	0.040	0.36 (0.12−1.04)	0.056
	KDIGO ≥ 2^a^	2.81 (1.19−6.61)	0.016	2.63 (1.07−6.53)	0.037
	Ejection fraction				
	≥ 50%	Ref.		Ref.	
	50% to ≥ 40%	1.20 (0.54−3.10)	0.520	1.17 (0.27−4.68)	0.835
	< 40%	1.53 (0.91−6.08)	0.066	1.49 (0.64−5.52)	0.320
	Inotropes use	2.33 (0.89−6.83)	0.082	1.96 (0.66−5.84)	0.228
CKD	Age^b^	1.09 (1.04−1.14)	< 0.001	1.10 (1.05−1.15)	< 0.001
	Hypertension	2.73 (1.19−6.26)	0.016	1.91 (0.77−4.75)	0.162
	Creatinine peak^b^	1.48 (1.06−2.08)	0.012	1.82 (1.06−3.12)	0.030

^a^ KDIGO severity 2 and above include a serum creatinine of 2.0 and above multiplied by baseline.

^b^ Odds ratio for every one additional year of age.

^c^ Odds ratio for every additional 1 mg/dL of creatinine.

Among subjects with CKD, univariate analysis found older age, hypertension, and higher peak creatinine to be associated with AKD (Table 2). In multivariate analysis, older age (AOR for every additional year 1.09, 95% CI 1.04−1.14, *p* < 0.01) and peak creatinine (AOR for every 1 mg/dL increase in creatinine 1.48, 95% CI 1.06−2.08, *p* = 0.03) remained independent predictors.

### 3.1 AKD as a Prognostic Marker

**Figure 2:**
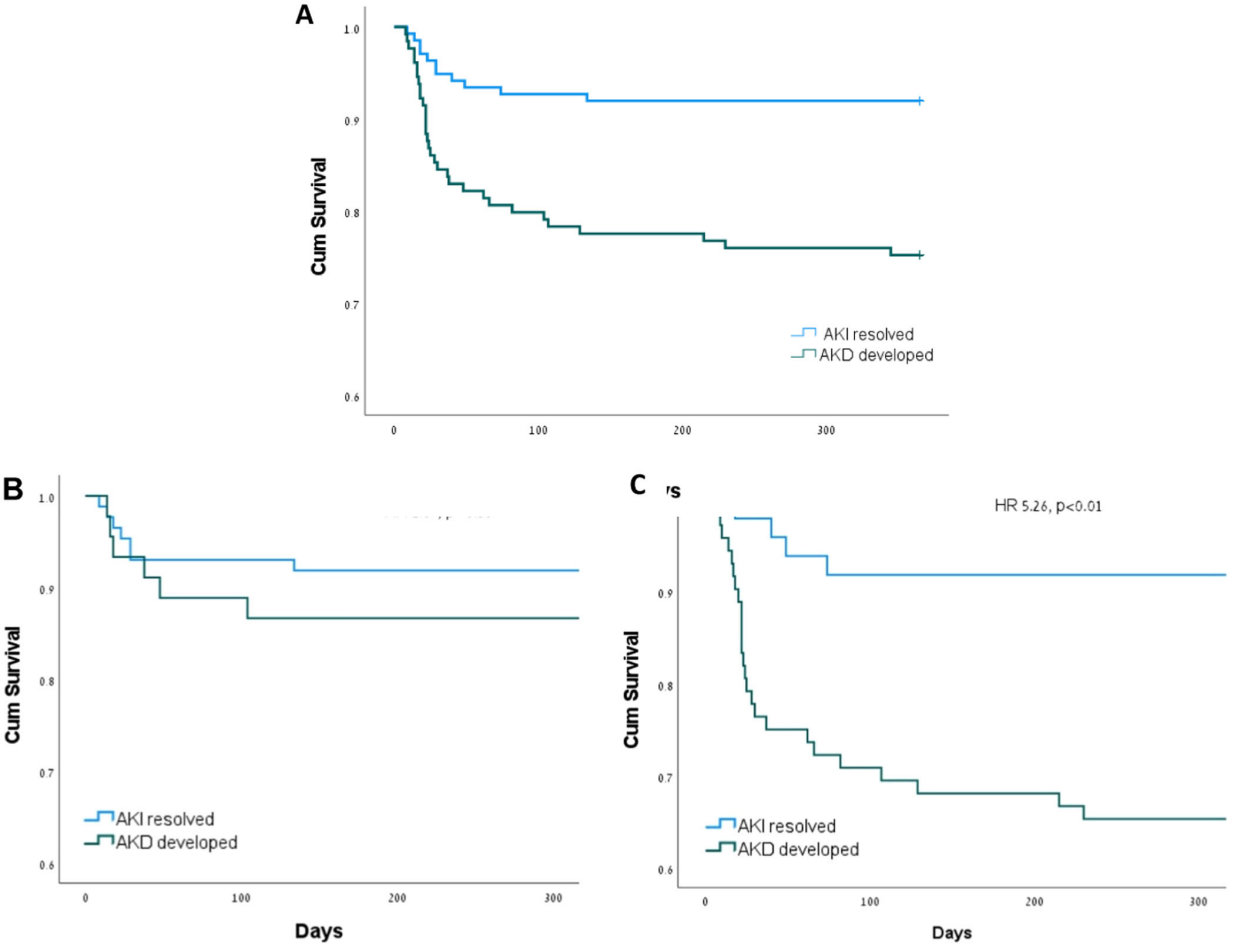
Kaplan−Meier curve for survival based on the development of AKD, among all patients (A) patients without CKD (B) and with CKD (C).

In our study cohort, AKD was a risk factor for worse 1‐year survival (HR 3.39, 95% CI 1.71−6.72, *p* < 0.01), as also presented in Figure 2. However, in a subgroup analysis, the higher mortality was observed only in subjects with CKD (HR 5.26, 95% CI 1.83−15.1, *p* < 0.01) and not in subjects without (HR 1.67, 95% CI 0.56−4.96, *p* = 0.36).

## 4 Discussion

This study aimed to evaluate the prevalence and predictors of AKD among STEMI patients who developed AKI. Among these patients, almost 50% progressed to AKD, with even higher prevalence in patients having CKD. Furthermore, AKD was associated with higher rates of mortality at 1 year follow‐up for CKD patients alone. Regarding AKD predictors, different features were found associated with AKD based on the presence of CKD at baseline, suggesting different mechanism in play, as discussed below.

Renal injury is a common complication of ACS patients in general and of STEMI patients in particular, with many reports of significant correlation to adverse outcomes and mortality [15, 16]. Yet, AKI and CKD are traditional definitions that lack adequate stratification for patient who are at risk for renal deterioration or other adverse outcome [17]. Later studies investigated renal recovery as an important feature and found that early recovery may reflect lower risk for adverse outcomes [10, 11, 16]. This has led to an updated definition of renal injury with delayed recovery (> 7 days), that is, AKD [14].

Being a relatively new definition, there is limited epidemiological data regarding the prevalence of AKD, especially in the setting of acute cardiac care. Most studies performed used the term “transient renal injury” loosely, setting end points for renal recovery ranging from 1 month to 1 year [18, 19]. However, a recent study found up to 30% of patients with AKI after an urgent coronary intervention developed AKD [9]. We found even higher rates, with 46% of patients with AKI who progressed to AKD. This difference can be largely attributed to methodology differences, as we measured our outcome after Day 7 while said report waited 30 days for their primary outcome. In addition, our population consisted solely from STEMI patients which are much more prone to persistent renal injury given the nature of the initial renal insult and prolonged hospitalization [2].

As mentioned earlier, STEMI patients are highly susceptible to AKI with numerous mechanisms offered, including hemodynamic instability, contrast media exposure, the use of nephrotoxic medication, and residual reduced ejection fraction [2, 20]. However, renal recovery in this subset of patients gained interest in recent studies as it was found to be associated with favorable outcomes [21, 22]. These factors are known to be associated with AKI. Whether they also lead to long‐term kidney dysfunction is not clear. For example, previous research have shown that renal recovery in STEMI patients was not lower in subjects that underwent mechanical ventilation [23, 24]. It was hypothesized that the initial insult of hemodynamic compromise associated with STEMI or the above complications has little impact upon renal recovery. Our results lay in adherence with these previous reports, as features linked to hemodynamic instability and reduced renal perfusion (Ionotropic use and ejection fraction) were initially significant, yet failed to remain predictive upon multivariate analysis.

When performing advanced analysis, we found that higher KDIGO stage (≥ 2) was the only factor independently associated with AKD in non‐CKD patients. These results are not surprising as the continuum of KDIGO staging was associated with adverse outcomes in numerous reports [25, 26]. However, given our results, STEMI patients with KDIGO ≥ 2, who have been exposed to contract media, have unknown residual cardiac function and expected to receive a variety of nephrotoxic medication—require special care and possible intervention. One example of such intervention can be found in recent studies offering care bundles for STEMI patients to reduce renal injury and improve recovery [24, 27].

Gender was previously described to be associated with various post‐intervention complication including AKI [28]. On the contrary, we found female gender to be borderline protective for delayed recovery and AKD formation. We hypothesize that much like hemodynamic instability discussed above, females were more prone to AKI due to delayed presentation and added baseline features associated with AKI. However, these factors do not necessarily effect renal recovery. In our cohort, after adjusting for comorbidities by the regression model, female gender became associated with better results, as also shown in some previous studies [29].

Sharma et al. reviewed the topic of renal functional reserve (RFR) in their article from 2014. They raised the possibility that in the presence of low RFR, renal recovery will be limited [30]. Indeed, while attempting to further divide our cohort population into preexisting renal disease we found that CKD patients have different risk factor for AKD, age being the most prominent one. Yet, age by itself was not found associated with AKD in non‐CKD patients. These results lay in adherence with previous reports suggesting age as a dominant factor in persistent renal damage [9, 18]. Sharma et al. notion, that the combination of CKD and older age represent lower “renal reserve”—making recovery from AKI less likely, is also supported by our results.

Our study bears some notable limitations. First, being a single center study, the generalizability of our results may be limited. Second, our follow‐up time for renal outcomes was relatively short and is not sufficient to determine progression to CKD or other long term renal outcomes. Third, we only followed on creatinine levels in patients with AKI during admission, yet AKD could initiate after discharge, possibly leading to underestimation of its general prevalence in our study. Long term follow‐up was performed in an outpatient setting resulting in lack of available data for analysis regarding long term renal outcomes or mortality etiology. Further studies with longer and structured follow‐up plan are needed to provide insights regarding the clinical significance and renal morbidity of AKD.

In conclusion, AKD is common among STEMI patients with subsequent AKI. CKD and higher KDIGO stage should prompt strict monitoring for early diagnosis, treatment, and prevention of prolonged renal function deterioration.

## Author Contributions

**Shir Frydman:** writing–original draft, data curation. **Ophir Freund:** statistical analysis, writing–original draft. **Haytham Abu Katash:** writing–review and editing. **Daniel Rimbrot:** data curation. **Shmuel Banai:** methodology, writing–review and editing. **Yacov Shacham:** conceptualization, methodology, writing–review and editing.

## Ethics Statement

Study protocol was reviewed and approved by the institutional ethics committee. The study was conducted in accordance with the standards as laid down in the in the 1964 Declaration of Helsinki and its later amendments.

## Consent

Informed consent was obtained by writing from all participants.

## Conflicts of Interest

The authors declare no conflicts of interest.

## Data Availability

The data that support the findings of this study are not publicly available due to containing information that could compromise the privacy of research participants but are available from the corresponding author, S.F., upon reasonable request.
